# Prognostic Significance of Abnormal Ankle–Brachial Index Among Long-term Hemodialysis Patients in Kinshasa, the Democratic Republic of the Congo

**DOI:** 10.5041/RMMJ.10427

**Published:** 2021-01-19

**Authors:** Yannick Mompango Engole, François Bompeka Lepira, Yannick Mayamba Nlandu, Yves Simbi Lubenga, Clarisse Nkondi, Augustin Luzayadio Longo, Aliocha Nkodila, Jean-Robert Rissassy Makulo, Vieux Momeme Mokoli, Justine Busanga Bukabau, Marie France Ingole Mboliasa, Evariste Mukendi Kadima, Cedric Kabemba Ilunga, Chantal Vuvu Zinga, Nazaire Mangani Nseka, Ernest Kiswaya Sumaili

**Affiliations:** 1 Division of Nephrology-Dialysis, University of Kinshasa Hospital, Kinshasa, Democratic Republic of the Congo; 2 Division of Cardiology, University of Kinshasa Hospital, Kinshasa, Democratic Republic of the Congo; 3 City of the Blind Medical Center, Kinshasa, Democratic Republic of the Congo

**Keywords:** Ankle-brachial index, hemodialysis, subclinical peripheral arterial disease

## Abstract

**Objective:**

Early identification of atherosclerosis using a non-invasive tool like ankle–brachial index (ABI) could help reduce the risk for cardiovascular disease among long-term hemodialysis patients. The study objective was to assess the frequency and impact of abnormal ABI as a marker of subclinical peripheral artery disease (PAD) in chronic hemodialysis patients.

**Methods:**

This was a historic cohort study of kidney failure patients on long-term hemodialysis for at least 6 months. The ABI, measured with two oscillometric blood pressure devices simultaneously, was used to assess subclinical atherosclerosis of low limb extremities. Abnormal ABI was defined as ABI <0.9 or >1.3 (PAD present). Survival was defined as time to death. Independent factors associated with abnormal ABI were assessed using multiple logistic regression analysis. Kaplan–Meier method (log-rank test) was used to compare cumulative survival between the two groups; a *P* value <0.05 was statistically significant.

**Results:**

Abnormal ABI was noted in 50.6% (*n*=43) of the 85 kidney failure patients included in the study; 42.4% (*n*=36) had a low ABI, and 8.2% (*n*=7) had a high ABI. Factors associated with PAD present were cholesterol (adjusted odds ratio [AOR], 1.02; 95% confidence interval [CI], 1.01–1.04; *P*=0.019), inflammation (AOR, 9.44; 95% CI, 2.30–18.77; *P*=0.002), phosphocalcic product (AOR, 6.25; 95% CI, 1.19–12.87; *P*=0.031), and cardiac arrhythmias (AOR, 3.78; 95% CI, 1.55–7.81, *P*=0.009). Cumulative survival was worse among patients with PAD present (log-rank; *P*=0.032).

**Conclusion:**

The presence of PAD was a common finding in the present study, and associated with both traditional and emerging cardiovascular risk factors as well as a worse survival rate than patients without PAD.

## INTRODUCTION

Peripheral artery disease (PAD) of the lower extremities, an important manifestation of systemic atherosclerosis,^1^ is commonly seen in patients with kidney failure undergoing long-term hemodialysis (LTHD). In fact, the prevalence of PAD in LTHD patients has been reported to be high, ranging from 17% to 48%, and associated with increased cardiovascular morbidity and mortality.^2^,^3^ Peripheral artery disease shares similar risk factors with coronary artery disease and cerebrovascular disease.^3^ Therefore, its early diagnosis and management can help improve the prognosis of LTHD patients^4^ by avoiding or at least delaying adverse events, such as amputations, cardiovascular events, and death.^5^ In this regard, the ankle–brachial index (ABI) and pulse wave velocity are common non-invasive tools used to assess arterial health quantitatively with regard to blocked arteries and arterial stiffness, respectively.^6^ A low ABI has been reported to predict the future risk of cardiovascular disease and influence outcomes among LTHD patients.^7^

In the Democratic Republic of the Congo, very few studies have been carried out on the prevalence and prognostic significance of cerebral and cardiac diseases among LTHD patients,^8^,^9^ and data on the prevalence and prognostic significance of PAD in LTHD patients are not yet available. Therefore, the present study aimed to assess the burden and the prognostic significance of PAD among LTHD patients in Kinshasa.

## PATIENTS AND METHODS

### Study Population and Design

We conducted a historic cohort study that included all patients who attended four hemodialysis centers (University Hospital of Kinshasa, Medical Center of Kinshasa, Afia Medical Care, Ngaliema Medical Center) in Kinshasa, from March to December 2016. The patients had undergone hemodialysis two or three times a week with high-flux dialyzers, at a blood flow rate of 250–300 mL/min and dialysate flow rate of 500 mL/min, during each four-hour dialysis session. Patients aged 18–75 years who underwent long-term hemodialysis (LTHD) for at least 6 months were recruited. Exclusion criteria were atrial fibrillation, bilateral below the knee amputations, and recent hospitalization (less than 4 weeks prior to study enrollment). The study protocol was approved by the Ethics Committee of Kinshasa School of Public Health/University of Kinshasa (ESP/CE/013/2016), and written informed consent was obtained from all patients. All clinical investigations were conducted according to the principles expressed in the Declaration of Helsinki.

### Data Collection and Procedure

Information on demographic and medical data including sex, age, smoking history (ever versus never), kidney failure complications (encephalopathy, pericarditis, hypervolemia, acidosis, anemia, hypocalcemia), comorbidities (stroke and transient ischemic attack history, arrhythmias, heart failure, coronary artery disease, viral hepatitis C, human immunodeficiency virus, diabetes, hypertension), and dialysis parameters (number of sessions, vascular access, urea clearance, interdialytic weight gain) were obtained from interviews and the patients’ medical records. Body mass index was calculated as weight divided by height squared in kg/m^2^. Hypertension was defined as blood pressure (BP) ≥140/90 mmHg or taking antihypertensive drugs, and diabetes was defined based on a fasting blood glucose level of ≥126 mg/dL or taking antidiabetic drugs. Patients with a history of cerebrovascular accidents, including cerebral bleeding and infarction, were defined as having a cerebrovascular disease, and those with a history of angina or myocardial infarction, or ischemic changes in electrocardiography, were defined as having coronary artery disease. Laboratory parameters (blood urea nitrogen, serum creatinine, serum potassium, uric acid, bicarbonate, calcium, phosphorus, vitamin D, parathormone, hemoglobin and hematocrit, C-reactive protein, albumin and total protein, alkaline reserve, troponin, ProBNP) obtained one month or less following enrollment were retrieved from patients’ medical records.

### Evaluation of Cardiac Structure and Function

Echocardiographic examination was performed by an experienced cardiologist using a VIVID 7 system (General Electric Medical Systems, Milwaukee, WI, USA), with the patient breathing quietly while lying in the left lateral decubitus position. The cardiologist was blinded to other data. Two-dimensional M-mode images were recorded from the standardized views. Echocardiographic measurements included left ventricular internal diameter at diastole, the left ventricular posterior wall thickness at diastole, interventricular septal wall thickness at diastole, E-wave deceleration, and peak early and late diastolic transmittal filling velocity.

### Measurement of ABI

Since ABI may be influenced by hemodialysis,^10^ all ABI values were obtained by a trained and experienced physician 10–30 minutes before hemodialysis and after 5 minutes’ rest in the supine position. The ABI values were measured once in each patient using the Bidop ES-100 V3 arterial doppler device (Hadeco, Kawasaki, Japan), which automatically and simultaneously acquires oscillometric BP measurements in both arms and ankles.^11^ Occlusion and monitoring cuffs were placed tightly around the upper arms and both sides of the lower extremities in the supine position. Measurements were obtained from the posterior tibial arteries in the lower extremities, since the pedis dorsal artery is congenitally absent in 4% to 12% of the population.^12^ Systolic BP (SBP) was measured twice at each site, in rapid succession and alternating, to obtain an average value.^10^ Using the SBP ankle value the ABI was calculated as the ratio of *ankle SBP*/*arm SBP*. The ABI values were defined as follows: PAD present, abnormal ABI (<0.9 or >1.3) and PAD absent, normal ABI (0.9–1.3).

### Statistical Analysis

Statistical analysis was performed using SPSS, version 21. Continuous variables were expressed as mean ± standard deviation (SD) (normal distribution) or median and range (skewed distribution). Categorical variables were expressed as absolute (*n*) and relative (in percent) frequencies. Comparison of the means of two groups or more was done using Student’s *t* test or one-way analysis of variance (ANOVA) with Scheffé’s multiple test, respectively. Logistic regression analysis was used to assess independent factors associated with PAD. The endpoint was survival (time-to-death): the data of surviving patients at the end of the study (December 2019), patients lost to follow-up, or patients shifted to transplantation or peritoneal dialysis were also analyzed in the study. Kaplan–Meier analysis was used to describe survival, and the comparison of different survival rates was done using the log-rank test; statistical significance was defined as *P*<0.05.

## RESULTS

### General Characteristics of the Study Population

Sociodemographic and clinical characteristics of the study population are given in Table 1. Eighty-five patients (67% men) were enrolled in the study; mean age was 52.8±15.9 years, with more than half aged 60 years or younger. Average values for body mass index, waist circumference, SBP, diastolic BP (DBP), pulse pressure, and ABI were 25.2±5.2 kg/m^2^, 89.8±16.1 cm, 156.2±18.7 mmHg, 84.5±15.3 mmHg, 71.7±17.9 mmHg, and 1.01±0.2, respectively. Hypertension (*n*=78, 91.8%) and diabetes (*n*=32, 37.6%) were the main traditional cardiovascular risk factors observed. Their treatment was based on renin angiotensin system inhibitors (61.2%), calcium channel inhibitors (75.3%), diuretics (54.1%), antiplatelet aggregation (43.5%), antidiabetics (11.8%), and statins (14.1%).

**Table 1:** Sociodemographic and Clinical Characteristics of the Study Population as a Whole and According to ABI Status.

Characteristic	Overall (*n*=85)	Abnormal ABI (*n*=43)	Normal ABI (*n*=42)	*P*
ABI	1.01±0.2	0.98±0.24	1.04±0.15	0.202
Abnormal ABI, *n* (%)		43 (50.6)		
High		7 (8.2)		
Low		36 (42.4)		

Age, years	52.8±15.9	56.4±14.4	49.1±16.6	0.034
Age category, *n* (%)				0.047
<60 years	50 (58.8)	21 (48.8)	29 (69.0)	
≥60 years	35 (41.2)	22 (51.2)	13 (31.0)	

Alcohol, *n* (%)	23 (27.1)	10 (23.3)	13 (31.0)	0.290

Arrhythmia, *n* (%)	4 (4.7)	3 (7.0)	1 (2.4)	0.317

Body mass index, kg/m^2^	25.2±5.2	25.1±4.5	25.4±5.9	0.791

Blood Pressure, mmHg				
Diastolic	84.5±15.3	80.4±15.9	88.7±13.7	0.013
Systolic	156.2±18.7	154.7±21.9	157.7±14.9	0.458

Diabetes, *n* (%)	32 (37.6)	20 (46.5)	12 (28.6)	0.069

Gender, *n* (%)				0.561
Female	28 (32.9)	14 (32.6)	14 (33.3)	
Male	57 (67.1)	29 (67.4)	28 (66.7)	

Gout, *n* (%)	11 (12.9)	5 (11.6)	6 (14.3)	0.483

Heart failure, *n* (%)	20 (23.5)	11 (25.6)	9 (21.4)	0.423

Hypertension, *n* (%)	78 (91.8)	38 (88.4)	40 (95.2)	0.226

Inflammation, *n* (%)	56 (65.9)	34 (79.1)	22 (52.4)	0.009

Medications, *n* (%)				
Antiarrhythmic	3 (3.5)	2 (4.7)	1 (2.4)	0.509
Aspirin	37 (43.5)	16 (37.2)	21 (50.0)	0.166
Calcium channel inhibitors	64 (75.3)	32 (74.4)	32 (76.2)	0.525
Diuretics	46 (54.1)	24 (55.8)	22 (52.4)	0.460
Insulin	11 (12.9)	6 (14.0)	5 (11.9)	0.517
Oral antidiabetic	10 (11.8)	8 (18.6)	2 (4.8)	0.048
RAS inhibitors	52 (61.2)	30 (69.8)	22 (52.4)	0.504
Statin	12 (14.1)	5 (11.6)	7 (16.7)	0.362

MICS, *n* (%)	32 (37.6)	16 (37.2)	16 (38.1)	0.555

Obesity, *n* (%)	10 (11.8)	4 (9.3)	6 (14.3)	0.354

Pulse pressure, mmHg	71.7±17.9	74.2±19.9	69.1±15.3	0.186

Smoking, *n* (%)	10 (11.8)	3 (7.0)	7 (16.7)	0.147

Stroke, *n* (%)	10 (11.8)	5 (11.6)	5 (11.9)	0.616

Waist circumference, cm	89.8±16.1	93.5±14.1	86.9±17.2	0.119

Data are expressed as mean±standard deviation, absolute (*n*) and relative (in percent) frequencies.

ABI, ankle–brachial index; MICS, malnutrition inflammation complex syndrome; abnormal ABI (<0.9 or >1.4); normal ABI (0.9–1.3); RAS, renin angiotensin system.

Table 2 summarizes kidney failure and chronic hemodialysis characteristics of the study population. Table 3 presents the biological parameters of the study population.

**Table 2:** End-stage Renal Disease and Hemodialysis Characteristics of the Study Population as a Whole and According to Ankle–Brachial Index (ABI) Status.

Characteristic	Overall (*n*=85)	Abnormal ABI (*n*=43)	Normal ABI (*n*=42)	*P*
Hemodialysis sessions/week, *n* (%)				0.752
3 times	36 (42.4)	18 (41.9)	18 (42.8)	
2 times	39 (45.9)	21 (48.8)	18 (42.9)	
1 time	10 (11.8)	4 (9.3)	6 (14.3)	

Geriatric nutritional risk index	101.3±13.6	100.7±11.9	102.0±15.2	0.653

Hemodialysis duration, month	10.0 (5.0–18.5)	10.0 (5.0–19.0)	10.5 (5.0–18.3)	0.870

Initial nephropathy, *n* (%)				0.564
Chronic glomerulonephritis	25 (29.4)	11 (25.7)	14 (33.3)	
Diabetic nephropathy	30 (35.3)	17 (39.5)	13 (31.0)	
Nephroangiosclerosis	23 (27.1)	10 (23.3)	13 (31.0)	
Others	7 (8.3)	2 (4.7)	0 (0.0)	

Interdialytic weight gain, kg	1.96±0.83	1.79±0.78	2.1±0.9	0.050

Medications, *n* (%)				
Ca/Vit D supplement, *n* (%)	45 (52.9)	23 (53.5)	22 (52.4)	0.546
Erythropoietin, *n* (%)	62 (72.9)	34 (79.1)	28 (66.7)	0.149
Iron, *n* (%)	67 (78.8)	36 (83.7)	31 (73.8)	0.197
Phosphorus binders, *n* (%)	11 (12.9)	6 (14.0)	5 (11.9)	0.517

MICS, *n* (%)	32 (37.6)	16 (37.2)	16 (38.1)	0.555

Residual diuresis, mL/day	490 (300–700)	470 (300–900)	495.0 (300–612.5)	0.704

Vascular access, *n* (%)				0.056
Arteriovenous fistula	3 (3.5)	2 (4.7)	1 (2.4)	
Permanent catheter	8 (9.4)	1 (2.3)	7 (16.7)	
Temporary catheter	74 (87.1)	40 (93.0)	34 (81.0)	

Weekly urea clearance	1.13±0.23	1.13±0.24	1.13±0.22	0.979

Data are expressed as mean±standard deviation, median (interquartile range), absolute (n) and relative (in percent) frequencies.

Ca, calcium; Abnormal ABI (<0.9 or >1.4); normal ABI (0.9–1.3); Vit D, vitamin D.

**Table 3:** Biological Parameters of the Study Population as a Whole and According to Ankle–Brachial Index (ABI) Status.

Parameter	Overall (*n*=85)	Abnormal ABI (*n*=43)	Normal ABI (*n*=42)	*P*
24 h Proteinuria, g	1.2 (0.9–1.9)	1.1 (0.7–1.9)	1.3 (0.9–1.9)	0.801
Albumin, g/L	36.3±5.8	36.0±5.6	36.5±6.0	0.711
Bicarbonate, mmol/L	19.2±4.2	18.4±4.8	19.9±3.5	0.124
Calcium, mg/dL	8.0±1.3	8.3±1.2	7.7±1.3	0.022
Cholesterol, mg/dL	175.6±36.5	183.8±35.5	167.8±36.2	0.040
Creatinine, mg/dL	9.8±3.5	10.1±3.9	9.4±3.2	0.391
CRP, mg/L	12.0 (48–24.0)	12.0 (7.0–25.0)	11.0 (4.0–24.0)	0.160
Ferritin, ng/mL	470 (258.5–1335.5)	465.7 (263–1200)	613.8 (195.9–1861.6)	0.725
Glucose, mg/dL	116.2±44.8	116.6±29.8	115.7±56.7	0.923
HDL-c, mg/dL	46.9±18.5	45.2±19.4	48.6±17.7	0.404
Hematocrit, %	27.1±4.8	27.4±4.8	26.7±4.9	0.527
Hemoglobin, g/dL	8.9±1.5	9.0±1.4	8.8±1.5	0.461
Iron, μmol/L	21.0 (12.1–36.3)	21.0 (13.0–34.0)	17.9 (1 1.1–41.3)	0.659
LDL-c, mg/dL	107.8±33.5	113.2±34.2	102.6±32.4	0.149
MDRD-GFR, mL/min/1.73 m^2^	6.4±2.6	6.2±2.8	6.6±2.4	0.470
Parathormone, pg/mL	623.9±219.2	589.5±235.2	659.3±198.2	0.144
Phosphocalcic product, mg^2^/dL^2^	43.3±13.8	46.6±14.7	39.9±12.2	0.025
Phosphorus, mg/dL	5.4±1.7	5.6±1.8	5.2±1.5	0.217
Potassium, mmol/L	4.6±0.6	4.5±0.5	4.6±0.7	0.312
ProBNP, pg/mL	6265 (3409–21158)	5932 (2567–14100)	7322 (4116–22222)	0.179
Protein, g/L	66.5±9.8	66.9±9.4	66.1±10.4	0.675
Triglycerides, mg/dL	115.5±39.5	117.5±40.2	113.5±39.1	0.647
Troponin, ng/L	16.4 (6.9–32.0)	20.8 (8.0–33.0)	7.6 (3.7–7.6)	0.332
Uric acid, mg/dL	7.4±1.9	7.7±2.5	7.1±1.2	0.143
Vitamin D, ng/mL	23.9±3.5	24.4±4.5	23.6±1.9	0.328

Data are expressed as mean±standard deviation, median (interquartile range), absolute (*n*) and relative (in percent) frequencies.

CRP, C-reactive protein; HDL-c, high-density lipoprotein cholesterol; LDL-c, low-density lipoprotein cholesterol; MDRD-GFR, modification of diet in renal disease-glomerular filtration rate; Abnormal ABI (>0.9 or >1.4); normal ABI (0.9–1.3); ProBNP, pro-brain natriuretic peptide.

The echocardiographic and ABI parameters of the study population are summarized in Table 4.

**Table 4:** Echocardiographic and Ankle–Brachial Index Parameters of the Study Population as a Whole and According to ABI Status.

Variables	Overall (*n*=85)	Abnormal ABI (*n*=43)	Normal ABI (*n*=42)	*P*
Arrhythmia	21 (24.7)	16 (37.2)	5 (11.9)	0.006

Diastolic dysfunction, *n* (%)	71 (83.5)	35 (81.4)	36 (85.7)	0.404

Diastolic LVID, mm	50.0±6.0	50.2±6.7	49.9±5.3	0.834

Diastolic LVPW, mm	12.1±2.2	11.7±2.6	12.4±1.7	0.145

Early filling/atrial contraction	1.07±0.74	1.07±0.6	1.06±0.85	0.974

Early filling/E’	10.8±5.5	10.3±4.5	11.4±6.4	0.357

Inferior vena cava, mm	14.7±5.4	14.8±6.2	14.5±4.4	0.818

Interventricular septum, mm	13.2±2.3	12.7±2.2	13.8±2.2	0.031

LVH, *n* (%)	83 (97)	41 (95.3)	42 (100.0)	0.175
Concentric, *n* (%)	65 (76.5)	30 (69.8)	35 (83.3)	
Eccentric, *n* (%)	18 (21.2)	11 (25.6)	7 (16.7)	

LVMI, g/m^2^	368.7±148.4	349.7±176.5	388.1±111.8	0.236

LVEF, %	64.4±9.0	64.9±8.4	63.9±9.7	0.565

Systolic dysfunction, *n* (%)	73 (85.9)	39 (90.7)	34 (81.0)	0.164

Data are expressed as mean±standard deviation, absolute (*n*) and relative (in percent) frequencies.

ABI, ankle–brachial index; E’, the mean of the septal and lateral E wave in tissue Doppler at the mitral ring; LVEF, left ventricular ejection fraction; LVH, left ventricular hypertrophy; LVID, left ventricular internal diameter; LVMI, left ventricular mass index; LVPW, left ventricular posterior wall; Abnormal ABI (<0.9 or >1.4); normal ABI (0.9–1.3).

### Frequency and Clinical Profile of Abnormal ABI

Abnormal ABI was observed in 43 (50.6%) patients, of which 36 (42.4%) had a low ABI and 7 (8.2%) had a high ABI (Table 1). Compared to patients with a normal ABI, patients with an abnormal ABI were on average older (56.4±14.4 versus 49.1±16.6 years; *P*=0.034), and a significantly higher proportion were 60 years old or more (51.2% versus 31%; *P*=0.047). These patients also had, on average, significantly decreased DBP (80.4 mmHg versus 88.7 mmHg; *P*=0.013) and a significantly higher proportion of inflammation (79.1% versus 52.4%; *P*=0.009). Differences observed in other parameters of interest did not reach the level of statistical significance (Table 1). With reference to kidney failure and hemodialysis parameters, patients with an abnormal ABI tended to have a higher proportion with temporary catheter access at initiation of hemodialysis (93.0% versus 81.0%; *P*=0.056); however, the difference observed was not statistically significant. The two subgroups were similar for the other variables of interest (Table 2).

For biological variables (Table 3), patients with abnormal ABI had on average significantly increased phosphocalcic product (P×Ca) (46.6 mg^2^/dL^2^ versus 39.9 mg^2^/dL^2^; *P*=0.025) and higher cholesterol (183.8±35.5 mg/dL versus 167.6±36.2 mg/dL; *P*=0.040) than those with normal ABI (Table 3). With reference to echocardiographic parameters, patients with abnormal ABI had on average significantly reduced interventricular septum levels (12.7± 2.2 cm versus 13.8±2.2 cm; *P*=0.031) and a significantly higher proportion of arrhythmia (37.2% versus 11.9%; *P*=0.006) compared to patients with normal ABI (Table 4). Differences in other echocardiographic parameters were not statistically significant.

### Factors Associated with Abnormal ABI

**Figure 1:**
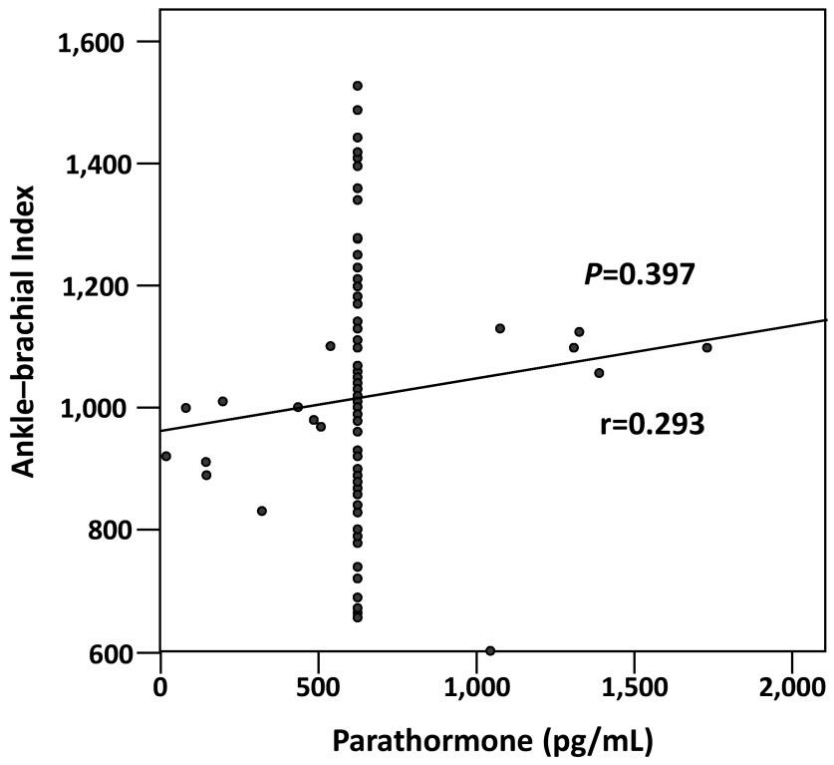
Correlation between Ankle-brachial Index and Parathormone.

Variables associated with abnormal ABI in univariate analysis were inflammation (*P*=0.011), arrhythmia (*P*=0.010), serum calcium (*P*=0.022), P×Ca (*P*=0.027), DBP (*P*=0.016), serum cholesterol (*P*= 0.047), and interventricular septum (*P*=0.035) (Table 5). In multivariate analysis (Table 5), the strength of the associations observed in univariate analysis persisted for inflammation (adjusted odds ratio [AOR] 9.44; 95% confidence interval [CI] 2.30–18.77; *P*=0.002), arrhythmia (AOR 3.78; 95% CI 1.53–7.81; *P*=0.009), cholesterol (AOR 1.02; 95% CI 1.01–1.04; *P*=0.019), and P×Ca (AOR 6.25; 95% CI 1.19–12.87; *P*=0.031). There was a positive correlation between parathormone and ABI, as demonstrated by the ABI curve increasing with the parathormone values, although the difference was not statistically significant (*r*=0.293, *P*=0.397) (Figure 1).

**Table 5:** Factors Associated with Peripheral Arterial Disease in the Study Population by Multiple Logistic Regression Analysis.

Variables	Univariate Analysis		Multivariate Analysis	

	*P*	Odds Ratio (95% CI)	*P*	Adjusted Odds Ratio (95% CI)
Inflammation				
No		1		1
Yes	0.011	3.43 (1.33–8.90)	0.002	9.44 (2.30–18.77)

Arrhythmia				
No		1		1
Yes	0.010	3.79 (1.38–10.39)	0.009	3.78 (1.53–7.81)

Phosphocalcic product, mg^2^/dL^2^				
≤55		1		1
>55	0.027	2.87 (1.91–9.02)	0.031	6.25 (1.19–12.87)

Diastolic blood pressure, mmHg*	0.016	1.04 (1.01–1.07)	0.202	1.03 (0.99–1.07)

Cholesterol*	0.047	1.01 (1.01–1.03)	0.019	1.02 (1.01–1.04)

Interventricular septum*	0.035	1.24 (1.02–1.52)	0.25	1.19 (0.88–1.62)

* Continuous variable.

### Abnormal ABI and Outcome

**Figure 2:**
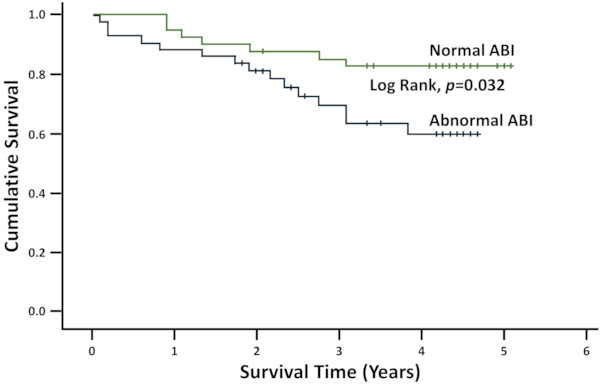
Cumulative Survival of the Study Population According to Ankle-brachial Index (ABI) Status.

During follow-up (5.0–18.5 months; mean=10 months), 22 (25.9%) patients died. Cumulative survival was better among patients with normal ABI (4.3 years; interquartile range [IQR] 4.2–4.5) compared with those with abnormal ABI (3.2 years; IQR 2.3–4.2) (log-rank; *P*=0.032) (Figure 2). Pulmonary arterial hypertension was found in 29% of patients with a mortality of 16% (4/25) of affected patients.

## DISCUSSION

The main findings of the present study are as follows. First, abnormal ABI (PAD present), mostly with a low ABI, was observed in half of the chronic hemodialysis patients. Second, PAD was associated with advanced age. Third, factors independently and significantly associated with PAD were P×Ca, arrhythmia, inflammation, and cholesterol. Fourth, cumulative survival was worse in patients with an abnormal ABI compared to those with a normal ABI.

Abnormal ABI was found in half of the chronic hemodialysis patients in this study, with a higher frequency than those reported by Tian (28%),^13^ Ašćerić (35%),^14^ and Ozgur (44%).^15^ Differences in sample size, study population characteristics, and criteria used to define abnormal ABI could explain the difference between their studies of abnormal ABI frequencies. Of note, due to financial constraints, most patients in our study had less than three dialysis sessions per week, resulting in accumulation of some uremic toxins, such as asymmetric dimethylarginine (ADMA), a well-known nitric oxide synthase inhibitor responsible for endothelial dysfunction and subsequent accelerated atherosclerosis.^16^

This study found that older age, especially >60 years, was associated with abnormal ABI, which is in agreement with previous reports by Ašćerić^14^ and Ozgur.^15^ The aging process could lead to accelerated atherosclerosis^17^ via vascular remodeling and insulin resistance, with a subsequent constellation of multiple cardiovascular risk factors, all of which have obesity in common as the main underlying factor.^18^,^19^ Hence, advanced age is clearly a risk factor for atherosclerosis, particularly if obesity is present.

Phosphocalcic product, inflammation, cholesterol, and arrhythmia emerged as the main independent factors positively associated with abnormal ABI in multivariate analysis. Our finding is consistent with that of previous reports of an association of traditional and emerging risk factors with abnormal ABI as a marker of atherosclerosis in the general population as well as in chronic hemodialysis patients.^10^,^20^,^21^ Increased calcium levels and phosphocalcic product, a marker of mediacalcosis, have already been reported in chronic hemodialysis patients with abnormal ABI or incompressible ankle by Miguel^10^ and Van Jaarsveld.^22^ Patients with abnormal ABI had increased levels of C-reactive protein as an inflammation biomarker in the present study. Our finding agrees with that of previous reports of an association of inflammation with accelerated atherosclerosis in chronic hemodialysis patients.^20^,^23^ There is a mutually triggering vicious cycle between inflammation and free radical production leading to oxidative stress and subsequent endothelial dysfunction and atherosclerosis. Indeed, proinflammatory cytokines can lead to excessive endothelial production of free radicals, and the latter can increase via activation of the nuclear factor kappa beta, the transcription of proinflammatory genes.^23^ Our finding of higher levels of total cholesterol and low-density lipoprotein cholesterol (LDL-c) in chronic hemodialysis patients with abnormal ABI is consistent with that reported by Jabbari.^7^ Increased cholesterol levels in chronic hemodialysis patients could be due to inflammation and uremic toxin-induced insulin resistance with subsequent lipid and glucose homeostasis disorders.^24^,^25^ In addition, since chronic hemodialysis is frequently associated with malnutrition, the latter could induce increased hepatic production of lipids due to the fall in oncotic pressure; such cholesterol levels are seen in nephrotic syndrome.^26^ In chronic hemodialysis, high levels of total cholesterol and LDL-c are associated with a high likelihood of carotid atheroma plaque formation.^27^,^28^ In addition to multiple traditional and emerging cardiovascular risk factors, the association of abnormal ABI with arrhythmia in chronic hemodialysis patients in the present study could be explained by the presence of valvular calcifications as reported by Ureña-Torres.^29^ Although vascular access failure has been reported to be frequently associated with abnormal ABI in chronic hemodialysis patients,^30^ the lack of association observed in the present study, where most patients used either temporary or permanent catheters, could be due to the small study sample size.

In the present study, survival was lowest among patients with an abnormal ABI, consistent with the findings of Miguel^10^ and Adragao^31^ who reported higher mortality rates among chronic hemodialysis patients with abnormal ABI.^32^ This could be explained by the fact that abnormal ABI is not only a marker of local endothelial dysfunction but also of extended endothelial dysfunction involving microcirculation of vital organs, such as heart, brain, kidneys, and lungs, which can lead to multiple organ failure.^3^ Therefore, abnormal ABI could be used for the prediction of global cardiovascular risk in chronic hemodialysis patients.

The interpretation of the results of the present study should take into account some limitations. First, the retrospective design of the present study precludes the establishment of any temporal relationship between exposure and outcomes. Second, the sample size did not allow sufficient power for statistical tests to identify potential relationships between variables of interest.

## CONCLUSION

Peripheral artery disease as assessed by ABI was a common finding in the present study and associated with both traditional and emerging cardiovascular risk factors as well as a low survival rate compared to patients without PAD. The validation of the present findings in a prospective study with a representative sample of chronic hemodialysis patients is planned.

## Abbreviations

ABI: ankle–brachial index
AOR: adjusted odds ratio
BP: blood pressure
CI: confidence interval
DBP: diastolic blood pressure
LTHD: long-term hemodialysis
PAD: peripheral arterial disease
P×Ca: phosphocalcic product
SBP: systolic blood pressure
